# Medical Opinion and Movement

**Published:** 1910-01-29

**Authors:** 


### Medical Opinion and Movement.

Dr. J. R. Hopkins, of Denver, has devised a new method of Treatment for the Condition of Surgical Shock. On the supposition that in surgical shock the peripheral blood-vessels are contracted, and those of the splanchnic area are dilated, the method consists in exciting the splanchnic nerves by means of a hot solution of normal saline injected into the abdominal cavity. The author gives details of a case in which this method was carried out with success. A woman aged 53 had undergone an operation of abdominal hysterectomy for a large uterine fibroma, and a few hours after the operation developed serious symptoms of shock. These symptoms increased in spite of the usual methods of treatment: elevation of the feet, repeated rectal injections of normal saline and coffee, and hypodermic injections of digitalis. The pulse became imperceptible at the wrist, and the breathing laboured and rapid. Dr. Hopkins then re-opened a small portion of the abdominal incision close to the umbilicus, and introduced a glass canula, connected by a rubber tube with a reservoir containing normal saline solution at a temperature of 44° C. The reservoir was held about 3 ft. above the abdomen, and the canula was carried upwards and backwards behind the transverse mesocolon to the region of the posterior wall of the stomach as near as possible to the solar plexus. About half a litre of the solution was allowed to flow in. For the first two or three seconds no pain was experienced, but soon afterwards the patient complained of acute pain due to irritation of the splanchnic nerves by the heat and pressure of the fluid. A sort of inverse shock was thereby produced. The radial pulse quickly returned strong and regular, and the patient completely recovered. The injection of the fluid only lasted a few seconds, the canula was quickly withdrawn, and the fluid retained by adhesive plaster and tight bandages. When one such injection fails to produce recovery the author advises a repetition in the course of an hour or so, and at the same time rectal injections of normal saline may also be resorted to.

---

An interesting article appears in the *Journal Médical Français*, by Drs. Paul Spillmann and Maurice Perrin, on the Ætiological Relationship of Syphilis to Tabes Dorsalis. The interest lies rather in the fact that the investigations of these authors fully confirm the close causal connection between the two diseases, as already shown by previous authorities, rather than in any new fact they disclose. Their observations embrace 105 cases of tabes, and in 81 of these a history of syphilis was admitted. In five others it was denied, but proved to have existed; in nine others it was probable, and in ten there was absence of evidence. These figures correspond almost exactly with those of other investigators, including Gowers, Erb, Raymond, Déjerine, and Strumpel. They also agree closely with those for general paralysis, and, as has been pointed out before, a higher percentage of positive evidence of syphilitic antecedents is not obtainable in cases of definite tertiary lesions. An analysis of the cases in regard to the interval of time between the incidence of the syphilitic attack and the onset of tabes shows also a close analogy to general paralysis. The larger proportion of cases occur between the sixth and fifteenth year, but one case occurred in the second year and one as late as 31 years afterwards. The third chief point in the analysis of these cases consists in the gravity of the syphilitic attack and the kind and duration of treatment undergone. Investigations under this head show that the attacks were of an ordinary nature and the treatment was always insufficient, and in 20 cases altogether absent. It is interesting to note in this regard that Dr. Spillmann has followed 32 cases of syphilis, rationally and thoroughly treated, for 15 years, and not one of them has developed tabes or general paralysis. Finally, a close analysis of the history of these 105 cases has failed to disclose any other ætiological factor which can even remotely compare with syphilis in constant relationship, such antecedents as alcoholism, stress and strain, hereditary nervous taint being only of rare occurrence.

---

Dr. Bettmann, writing in the *München Medizinische Wochenschrift* advocates the Internal Administration of Calcium Salts for the treatment of cases of dermatitis of toxic origin. He prescribes calcium lactate in 5 per cent. solution and orders one or two tablespoonfuls to be taken three times a day half an hour before meals. He has obtained success in many cases by this treatment without at the same time modifying the diet or habits of living of the patient. He has not seen much benefit result from the treatment in cases of purpura, except in one case, a man aged 32, who had had recurrent cutaneous hæmorrhages for over two years without any other apparent pathological lesion. Salicylates, arsenic, and other medicaments failed to have any effect, but the hæmorrhages completely ceased on the administration of calcium lactate for four weeks, and have not recurred since. Obstinate cases of urticaria have yielded to the treatment, and the author has found it especially efficacious in cases of senile pruritus.

---

A good deal has been heard of late of the Injection of Bismuth Paste into Tuberculous Sinuses both for therapeutic purposes and also as a means of examination and diagnosis by means of the *x*-rays. Dr. Leonard W. Ely gives a detailed account in the American *Journal of Surgery* of several cases treated in this way at the Sea Breeze Hospital, New York. The formula of Dr. Beck, of Chicago, was used—namely, 1 part of bismuth to 2 parts of vaseline. The mixture must be thoroughly homogeneous, and the addition of water must be carefully excluded. With this idea the glass syringe for injection should be boiled and then washed out with alcohol and the mouths of the sinuses wiped with alcohol. The mixture is heated in a water bath, and as soon as it is cooled sufficiently, so as to be only warm to the back of the hand, it is injected with as much force as the patient can tolerate. The injections are repeated about once a week. Altogether 14 cases of all kinds are reported, and of these five were cured, four improved, and five showed no improvement. The general conclusions from these cases are that the bismuth injections are of greater service in old infected sinuses than in recent ones, but they are of little value in the presence of visceral disease. The most remarkable case was that of a girl, aged 10, with lumbar Potts' disease and well-marked deformity. The disease was of eight years' duration, with suppurating sinuses for four years. There were one in each groin, one in the right buttock, and one over the left trochanter. Injections were begun in December 1908, and were followed by immediate improvement. The sinuses twice broke down again, but since May they have remained healed.

---

AT a recent meeting of the Académie de Médecine de Lille, Dr. Albert Robin read a paper confirming the fact which he had already established with Dr. Binat that in the case of Pulmonary Tuberculosis the respiratory interchanges are augmented in intensity. This augmentation is met with in all stages of the disease, even when the respiratory capacity is diminished as the result of the destruction of the pulmonary parenchyma. It is also met with in the descendants of the tuberculous and in those predisposed to the disease. The author once more insists on the importance, which he had already demonstrated, of the organic demineralisation which occurs in those predisposed and in the pretuberculous. He believes, therefore, that tuberculosis is the consequence of a hyperactivity of the organic functions.

---

PROFESSOR DASTRE has recently read an interesting paper before the Académie des Sciences embodying the results of a series of experiments carried out by Drs. Cuénot and Mercier, with reference to the effect of lactation on cancer in the mouse. The pregnant animals were inoculated with cancer, the disease following the usual course until characteristic lesions appeared. When, however, the animals entered on the period of lactation the cancerous growths rapidly retrogressed, and in some cases disappeared altogether, and a cure resulted. It would seem that a state of immunity to further cancerous infection was acquired, for the experimenters were unable to re-inoculate three mice in which the tumours had disappeared.

---

DR. ARCHONTAKIS, in a thesis recently published, again takes up the question of the semeiological value of the diminution of the vesicular murmur at the apex of the lung, a question which was discussed at length at a meeting of the Société Medicale des Hôpitaux. Bezançon, whose communication had been the starting-point of the discussion at that meeting, considers this diminution of the vesicular murmur as the indication of a healed tuberculous process. A large number of speakers recalled to mind a similar physical sign in certain cases suffering from lesions of the nasopharynx, adenopathies, thoracic deformity, functional spasm of the bronchi, chronic cardiopathy, localised emphysema at the apex of the lung, etc. Dr. Archontakis has submitted twenty patients showing this diminished vesicular murmur at the lung apex to radioscopic examination. Of these twenty patients four showed no other physical signs. In the rest a slight dulness of the suspected apex was found. In the four cases where a diminution of the vesicular murmur was the only anomaly found, a screen examination showed no modification of the apex. In the sixteen cases where the diminution was associated with slight dulness, a slight shadow was noticed at the suspected apex. The author concludes from this that diminution of the vesicular murmur is not by itself a sign of tuberculosis, but that, on the other hand, it indicates a bacillary infection when associated with other suspicious symptoms.

---

THERE is, says Sir Patrick Manson in the *British Medical Journal*, no anatomical or physiological reason for thinking that the European female is more susceptible to Trypanosome Infection than the European male. He points out that the white men exposed to this danger in Africa outnumber the women by at least 20 to 1—probably, we should say, by much more. Yet among the cases of trypanosomiasis in Europeans which Sir Patrick has had under observation about a third have been in females. This relatively enormous disproportion he judges to be no mere accidental coincidence, although as a matter of fact the number of his cases is not by any means large enough to exclude this possibility. Still he holds that there is a definite factor in the ætiology, and this he believes to be the usual feminine garb of petticoat and skirt. In three-fifths of the women suffering from trypanosomiasis whom he has seen the initial inoculation was through a bite on the leg. Skirts should therefore be abandoned, and a bloomer costume or loose trousers of some sort worn, securely tied round the top of the boots. Similarly the loose sleeves should be tied at the wrists. Sir Patrick condemns also the extensively abbreviated "shorts" largely affected by residents and sportsmen in tropical Africa. His views deserve to be widely circulated among those who may be intending to inhabit a tsetse-fly country.

---

A USEFUL summary of the latest researches into the Physiology and Pathology of Athletic Exercises appears in the *Medical Review*. According to Dr. Michell, of Cambridge, habitual athletics causes a progressive reduction of the pulse-rate to 48, or even 40, and a gradual increase in the size of the left ventricle. The cardiac first sound changes in character: the pitch is lowered, but the volume is increased and acquires a *crescendo* quality. The blood-pressure tends to rise, and the red corpuscles reach six millions or so, with a corresponding increase of the colour index. A fall in the red cell count and in the index is said to be the earliest sign of staleness in a trained man. Some of the signs of overwork of an athlete's heart are as follows: A rise in the morning pulse-rate before exercise. A longer persistence of the increased blood-pressure produced by exercise.' An equalisation of the intervals between the heart sounds. The position of the apex beat at the extreme point of the cardiac dulness. Another remarkable observation in these cases is a great sensitiveness of the heart's apex to pressure: the patient feels as if he would vomit if the stethoscope is pressed against the chest over the apex beat. Digitalis and strychnine are condemned by this author for such conditions. Restriction of the total quantity of food both liquid and solid; avoidance of tea, coffee, cocoa, and tobacco; rest; hot packs; and diuretin in 15-grain doses; all these are recommended.

---

THE completion of the fiftieth volume of the *Annals of Surgery* is celebrated in a double number full of interesting papers and more cosmopolitan than usual. Professor Harvey Cushing writes of Acromegaly and of Hypophysectomy for this disease. He begins with a note of warning as to the possible danger of removing the whole pituitary gland, on the analogy of what was found to happen when surgeons excised the whole of the thyroid. He points out that the operation has been done, and probably will be more often done in future, in conditions of simple hypophyseal hypertrophy without tumour. That is to say, acromegaly does not necessarily depend on a new growth in the pituitary body; and it is necessary, says the author, before any rational basis of treatment can be established, that a clearer understanding shall be gained of the clinical manifestations both of too much and too little pituitary substance. Much confusion still surrounds the relations of acromegaly, gigantism, and hypophyseal development; and even Marie, who first announced the connections between them, is still uncertain whether acromegaly is due to over-secretion or under-secretion of the gland. Dr. Cushing himself, as the result of animal experimentation, is inclined to associate adiposity plus infantilism of the sexual functions with lessened pituitary activity, and acromegaly and gigantism with hyper-pituitarism, often caused by an adenomatous condition of the gland. The explanation of the improvement that has often followed the removal of cysts, etc., is mechanical; that is, relief of pressure allows the remaining sound tissue once more to resume its functions.

---

BEYOND this, there is experimental evidence that the sudden removal in its entirety of the pars anterior (or epithelial lobe) of the hypophysis during health is incompatible with the maintenance of life; obviously this is a most important point in connection with the surgery of the region. These principles were applied practically to the case of a farmer, aged 38, who complained of frontal headache, photophobia, thickness of speech from an enlarged tongue, and increasing size of the jaw, hands, and feet. The latter symptoms had supervened very gradually over a period of several years. For two years the headache and other discomforts had been noticed. The mouth could be opened only 4 cm., and the lower teeth had been carried so far forward beyond the upper as to render it impossible for them to meet; the molars and the second bicuspids met, but not any anterior to this. After preliminary tracheotomy a partial hypophysectomy was done by the transphenoidal route with osteoplastic resection of the anterior walls of the frontal sinuses. The tracheal operation was done on account of the great difficulty occasioned by the enlarged tongue. There was no trouble with bleeding, as adrenalin solution was freely used throughout. About half the hypophysis was curetted away. Three days after the operation the patient got up; even before he left hospital a diminution in the size of his hands was evident, and later on measurements showed a definite, though not a great, improvement in the actual size of the bones. The one drawback was the loss of the sense of smell which occurred. Headache disappeared, but speech was not much improved. One precaution which is considered essential is the administration of urotropine beforehand, as this is followed by the appearance of formaldehyde in the cerebro-spinal fluid; thus a prophylactic against any possible meningeal infection is secured.

---

A SURVEY of 111 cases of Prostatic Cancer is contributed by H. Hampton Young, who draws the following conclusions, not very dissimilar to those of Mr. J. G. Pardoe reported a week ago in these columns. Carcinoma of the prostate accounts for about 20 per cent. of the cases of marked prostatic enlargement. It causes the same symptoms in the early stages as do simple hypertrophies; but later on neuralgic pain is often prominent, and often simulates that of other diseases. Carcinoma may begin as a small nodule in an otherwise healthy prostate, or in a chronic prostatitis. In cases of hypertrophy, malignant change (when it occurs) usually starts in a non-hypertrophied portion. The disease remains for a long time within the capsule of the gland; the urethra, bladder, etc., are invaded late. Such extra-prostatic invasion nearly always occurs first along the ejaculatory ducts into the space between the prostate, the vesiculæ seminales, and the bladder, spreading thence to the trigone. Any marked induration—even if only a small nodule—should be regarded with suspicion in a man over forty-five years of age, especially if there is no history of prostatitis; exploratory operation should always be advised in such cases, and should be conducted through the perineum. Should any attempt to deal thoroughly with the disease be practicable, the vesiculæ seminales, the vasa deferentia, and the anterior two thirds of the trigone should be removed as a routine measure. Cure has been known to result from this operation. Failing such possibilities, the interests of the patient are better served by a catheter life than by a suprapubic prostatectomy.

